# Pulmonary Valve Endocarditis during and beyond Euro ENDO Registry: A Single Center Case Series

**DOI:** 10.3390/medicina59071213

**Published:** 2023-06-28

**Authors:** Ilija Srdanović, Maja Stefanović, Tatjana Miljković, Snežana Bjelić, Miloš Trajković, Teodora Pantić, Lazar Velicki, Aleksandra Milovančev

**Affiliations:** 1 Faculty of Medicine, University of Novi Sad, 21000 Novi Sad, Serbia; ilija.srdanovic@mf.uns.ac.rs (I.S.); maja.stefanovic@mf.uns.ac.rs (M.S.); tatjana.miljkovic@mf.uns.ac.rs (T.M.); snezana.bjelic@mf.uns.ac.rs (S.B.); lazar.velicki@mf.uns.ac.rs (L.V.); 2 Institute of Cardiovascular Diseases of Vojvodina, 21204 Sremska Kamenica, Serbia; milos.trajkovic@ikvbv.ns.ac.rs (M.T.); teodora.pantic@ikvbv.ns.ac.rs (T.P.)

**Keywords:** pulmonary valve, infective endocarditis, endocarditis team, surgery

## Abstract

*Background*: Pulmonary valve infective endocarditis (PVIE) is a rare form of infective endocarditis (IE) and is associated with high mortality and severe complications. Guidelines for treatment of this form of IE are scarce and based on general recommendations. We report a case series of PVE. *Detailed Case Description*: Case 1—A 36-year-old female with congenital pulmonary artery stenosis, dyspnea and leg edema symptoms for 2 months. Blood cultures yielded *Staphylococcus* spp. and *Corynebacterium* sp., and echocardiography revealed multiple floating vegetation at the pulmonic valve and surrounding structures. The clinical course was complicated with sepsis and multi-organ failure. Urgent surgery with pulmonary homograft implantation resulted in successful five-year outcome. Case 2—In a 38-year-old male with previous tetralogy of Fallot correction and symptoms of fatigue, fever, myalgia, and photophobia, echocardiography was suggestive of PVIE. The clinical course was complicated with septic shock, multi-organ failure, ischemic stroke with hemorrhagic transformation and death on the 12th day of hospitalization. Case 3—A 41-year-old male without previous medical history was hospitalized due to prolonged fatigue, fever, dyspnea, and leg edema. He was diagnosed with multi-valve infective endocarditis, affecting the aortic, tricuspid, and pulmonary valve. Acute heart failure and hemodynamic instability indicated urgent surgery with aortic valve replacement and reconstruction of the tricuspid and pulmonary valves. At four-year follow up he was doing well. *Conclusion*: Symptoms in PVIE may be versatile, and diagnosis is often delayed. High level of suspicion, early recognition, and echocardiography are cornerstones in diagnostics. Despite the standpoint that medical therapy is first-line, the role of surgery needs to be advocated in particular cases.

## 1. Introduction

Infective endocarditis is rare but still associated with high morbidity and mortality. Right-sided IE (RSIE) accounts for 10% of all infective endocarditis (IE) cases [1], with pulmonary valve endocarditis (PVIE) being extremely rare and representing only 1–2% of all IE cases [2]. Some surgical series report 0.5% PVIE involvement incidence within 1362 operated IE patients [3]. Lower pressure gradients at the valves, combined with reduced blood oxygen within the right-heart chambers, might contribute to the development of RSIE [4]. Nowadays PVIE is most often seen in patients who have undergone surgical or percutaneous pulmonary valve (PV) procedures [5,6,7,8,9].

Initial PVIE treatment, as well as other RSIE treatments, are based on intravenous antibiotics; however, surgical intervention may be warranted in several situations, such as uncontrolled infection, huge embolic potential, and hemodynamic deterioration [10].

We report three cases of PV endocarditis. The aim of this case series is to describe underlying risk factors, symptoms, clinical and diagnostic challenges, microbiological features, and management of PVIE. These patients were part of the ESC-EORP Euro-ENDO registry where our high-volume center, among 156 others from 40 countries, recruited 42 (1.4%) patients [11].

## 2. Detailed Case Series Description

### 2.1. Case Report No 1

**Figure 1:**
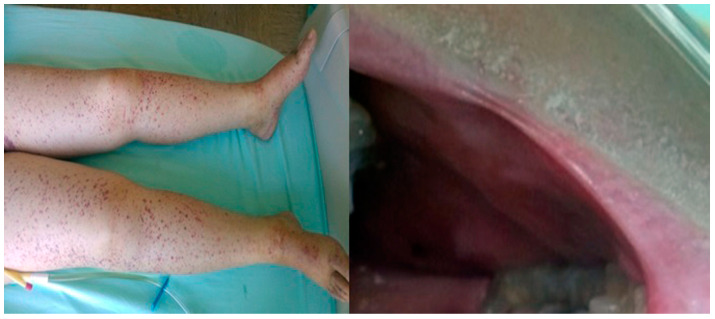
Skin hemorrhage at the lower extremities and in mouth mucosa—Case 1.

A 36-year-old female was hospitalized for symptoms of dyspnea and leg edema that started 2 months before. She suffered from adult congenital heart disease (ACHD)—pulmonary artery stenosis. According to data from the community care hospital, urine culture was positive for *Staphylococcus*. In-hospital trans-thoracic echocardiography (TTE) revealed multiple vegetations on the stenotic pulmonic valve. Repeated blood cultures were positive for *Staphylococcus* spp. and *Corynebacterium* sp. An antibiotic treatment with Vancomycin and Ciprofloxacin was introduced. Physical examination revealed leg edema and diffuse petechiae on the skin of the abdomen and legs, as well as the mouth mucosa (Figure 1).

**Figure 2:**
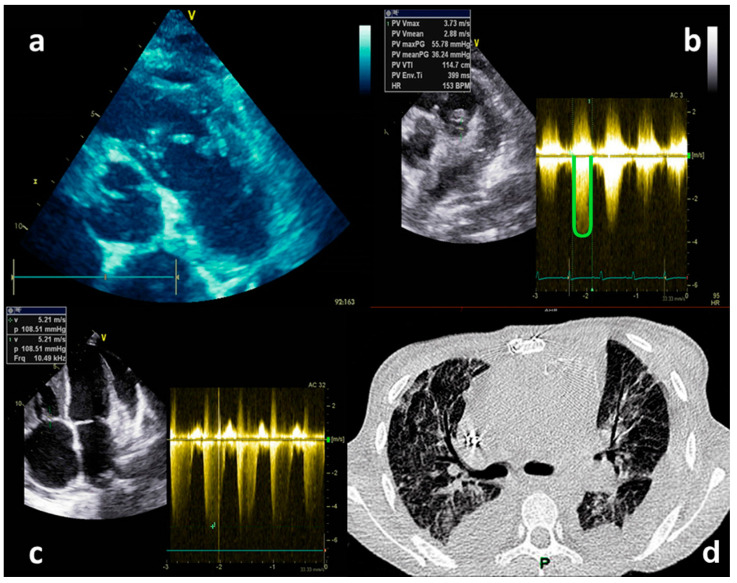
Echocardiography TTE and CT imaging−Case 1. (**a**) TTE−Multiple floating vegetations in the right ventricular outflow tract, pulmonary valve, and on the wall of the pulmonary artery. (**b**) TTE−Gradient across pulmonary valve. (**c**) TTE−Maximal velocity of the tricuspid regurgitation. (**d**) MSCT−Multiple septic embolizations in the lung parenchyma, lung atelectasis, pleural effusion.

**Figure 3:**
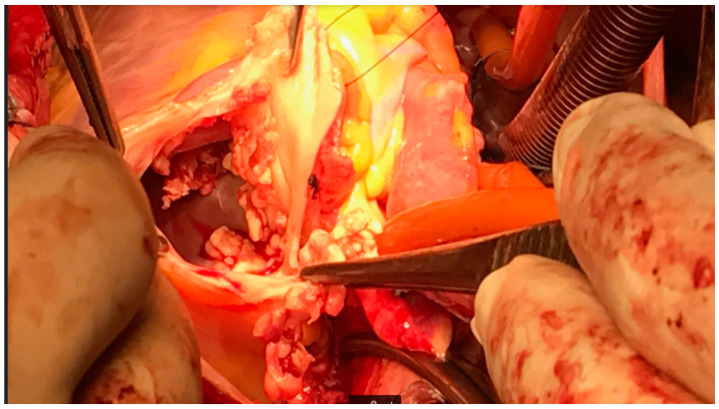
Intraoperative finding—Case 1. By courtesy of Prof. Darko Anic MD PhD.

Heart auscultation revealed predominantly systolic as well as diastolic murmur over the pulmonic valve, and lung auscultation was suggestive of pleural effusion due to absent breath sounds in basal and mid-lung portions. The laboratory findings show a leucocyte count of 10.72 [ref. 4–10] × 10^9^ hemoglobin of 79 [ref. 120–170] g/L, C-reactive protein (CRP) was 50 [ref. 0–5] mg/L, procalcitonin 0.96 [ref. 0.0–0.05] ng/mL, and creatinine [ref. 50–120], e GFR 40 mL/min/1.73 m^2^. A chest X-ray was suggestive of pleural effusion, showing homogenous opacity and obliteration of the costophrenic angles bilaterally. Diagnostic thoracocentesis with cytochemical analysis confirmed that the fluid was exudate. TTE revealed multiple floating vegetations (up to 2 cm) (Figure 2a) at the right ventricular outflow tract, pulmonary valve, and the walls of the pulmonary artery (Video S1: Vegetations on PV and RVOT), with significant pulmonary valve stenosis with a transpulmonary valve gradient of 55 mmHg (Figure 2b), and moderate pulmonary valve regurgitation. The ejection fraction (EF) of the left ventricle was preserved. The right heart chamber’s diameters were normal, tricuspid regurgitation was estimated as moderate and right ventricle systolic pressure was severely increased by 108 mmHg (Figure 2c). A Thoracic CT scan showed multiple septic embolizations in the lung parenchyma, lung atelectasis, pleural effusion, and multiple floating vegetations in the pulmonary artery (Figure 2d). Despite the optimization of antibiotic therapy (2 weeks of Vancomycin and Gentamycin with concomitant treatment), she remained with laboratory and clinical signs of sepsis, with persistently worsening renal failure. Repeated TTE was without changes in distribution and size of previously seen vegetations, but with signs of both left (drop in EF to 30%) and right ventricular failure. Our hospital’s endocarditis team (ET) decided to perform the surgery with homograft implantation. Since there were no homografts in our country, after the international ET evaluation, she underwent cardiac surgery in the neighboring country of Croatia. Her EuroSCOREII was 10.68%. On the pulmonary position, a homograft was placed, and a resection of stenotic and infected infundibulum was performed (Figure 3). The postoperative course went well. Postoperative TTE showed normal trans-pulmonic valvular gradients without regurgitation, left ventricular improvement from LVEF 35% to 50%, moderate tricuspid regurgitation, and one floating filamentous formation connected to the septal cusp of the tricuspid valve. After surgery, she has referred again to our hospital. Transesophageal echocardiography (TOE) revealed that the filamentous formation was a thrombotic mass. The treatment was continued with parenteral antibiotics and anticoagulant therapy. After 54 days postsurgery, TTE verified that the thrombus was dissolved. She was discharged in good condition on the 57th postoperative day. Five years later, she remains well, with no symptoms, and handling physical strains without problems.

### 2.2. Case Report No 2

**Figure 4:**
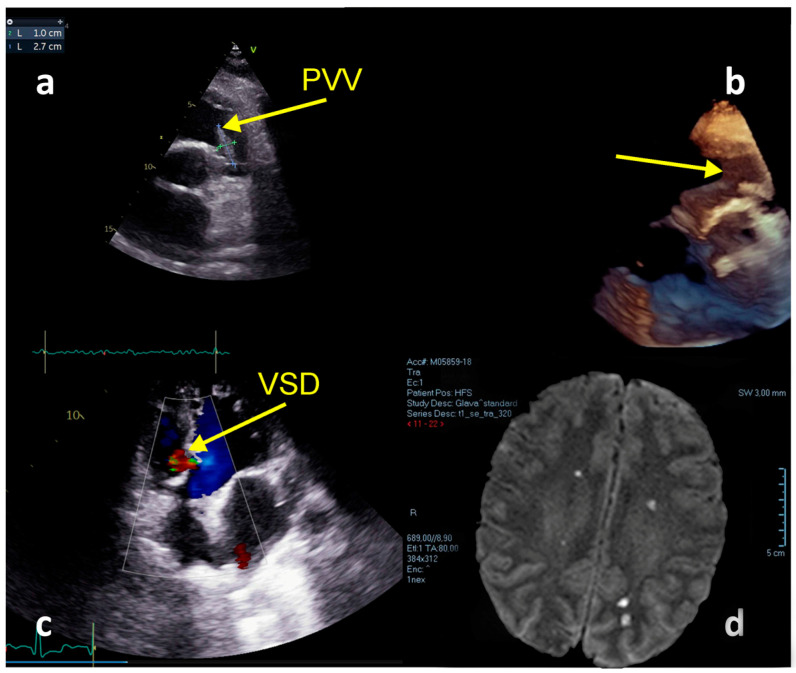
Echocardiography and MRI imaging—Case 2. (**a**) Floating vegetation in the RVOT. (**b**) TTE + 3D reconstruction. (**c**) Residual VSD; (**d**) MRI—Multiple septic brain embolization.

A male aged 38 years was referred to our hospital from the clinic for infectious diseases, with a diagnosis of severe sepsis. He experienced 5 days of symptoms of increased temperature up to 40 °C, fatigue, myalgia, and photophobia. An initial brain CT scan and ultrasonography of the abdomen revealed no significant changes. Antibiotic treatment was initiated with levofloxacin. After TTE suggestive of PVIE the patient was transferred to our hospital. His medical history included tetralogy of Fallot surgical repair at the ages of 1 and 7. At admission to our hospital, he had symptoms and signs of heart failure (HF) and multiple organ failure (MOF). A whole systolic murmur (4/6) was heard at the precordium. Laboratory tests at admission found a leucocyte count of 20.33 [ref. 4–10] × 10^9^, hemoglobin 117 [ref. 120–170] g/L, PLT 39 [ref. 150–400] × 10^9^, CRP 155 [ref. 0.0–0.05] mg/L, creatinine 416 [ref. 50–120] μmol/L; eGFR 15 mL/min/1.73 m^2^, NT-proBNP: >25,000 [ref. 0.0–125.0] pg/mL, procalcitonin: 50.68 [ref. 0.0–0.05] ng/mL, hs-Troponin: 151.4 [ref. 0.0–25.0] ng/L; D-Dimer: 5018.88 [ref. 0.0–500.0] ng/mL. TTE revealed massive PV vegetation (Video S2: Pulmonary valve vegetation) (dimension 2.4 × 0.7 cm), residual ventricular septal defect (VSD) in the membranous part of the interventricular septum, impaired left ventricular systolic function with EF of 25–30%, right ventricular enlargement with decreased systolic function (TAPSE = 1.5 cm; FAC = 43%; S’ = 0.08 m/s), and pulmonary hypertension of 43 mmHg (Figure 4a–c). He was treated with therapy attributed to heart and renal failure: diuretics, dobutamine, noradrenaline, and continuous venovenous hemodiafiltration (CVVHDF). During the hospitalization, several antibiotics were used (metronidazole, linezolid, ceftriaxone) and no positive blood cultures were found. On the first day after the admission, the hospital ET indicated PV homograft surgery. On the second hospital day, the patient experienced deep neurological deterioration. Magnetic resonance of the brain (Figure 4d) showed multiple septic embolizations. His EurSCOREII was 83.23%. Unfortunately, the lack of PV homograft and neurological complications led to postponing the cardiac surgery procedure and resulted in a lethal outcome on the 12th day of hospitalization, due to a hemorrhagic transformation of ischemic stroke.

### 2.3. Case Report No 3

A 41-year-old male was referred to our tertiary care hospital with confirmed IE of the aortic, tricuspid, and pulmonary valves, along with severe HF. He was previously hospitalized in secondary care hospital due to prolonged fatigue, fever, dyspnea, and leg edema. Initial treatment with antibiotics and supportive therapy did not yield any improvement. Laboratory findings at the admission to our hospital revealed a leucocyte count of 5.8 [ref. 4–10] × 10^9^, hemoglobin 93 [ref.120–170] g/L, PLT 218 [ref.150–400] × 10^9^, CRP 57.2 [ref.0–5] mg/L, creatinine 226 [ref.50–120] μmol/L; eGFR 31 mL/min/1.73 m^2^, NT-proBNP: >25,000 [ref.0–125] pg/mL, prokalcitonin: 0.15 [ref.0–0.05] ng/mL, hs-Troponin: 151.4 [ref.0–25] ng/L; D-Dimer: 5018.88 [ref.0–500] ng/mL. The blood culture samples yielded *Staphylococcus* and *Klebsiella pneumonia*, and treatment with susceptible antibiotics vancomycin and gentamicin was introduced.

**Figure 5:**
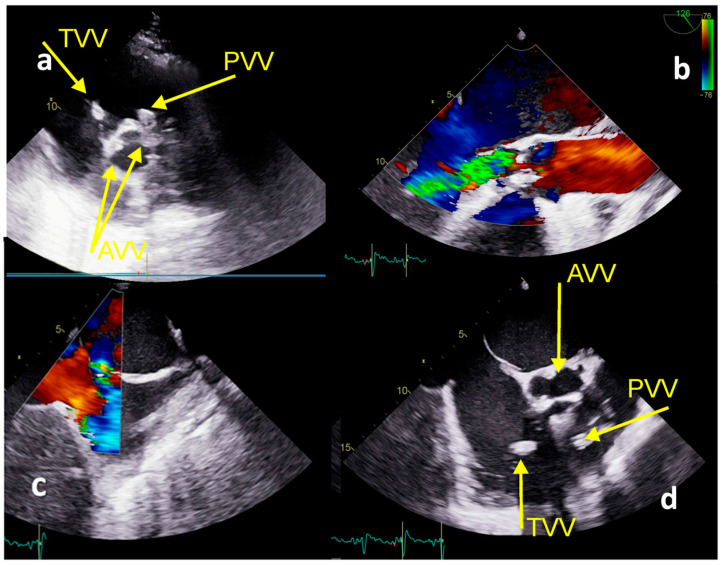
Echocardiography TTE and TEE imaging−Case 3. (**a**) TTE: TVV−vegetation at the tricuspid valve, PVV−vegetation at the pulmonary valve, AVV−vegetations at the aortic valve; (**b**) TOE−severe aortic regurgitation; (**c**) TOE−patent foramen ovale; (**d**) TOE−vegetations at the tricuspid, pulmonary, and aortic valves.

TTE and TOE confirmed floating vegetations at the tricuspid valve (1.4 cm × 1.3 cm) with severe regurgitation and right ventricular systolic pressure of 85 mmHg, PV with floating vegetation (1.5 cm × 1.0 cm) (Figure 5a,d), the destruction of the aortic valve with severe aortic regurgitation (Figure 5b), and patent foramen ovale with L-R shunt (Figure 5c), with left ventricular EF of 53%. Severe hemodynamic deterioration with pulmonary edema advocated emergency ET to indicate urgent surgery. His EuroSCOREII was 52.38%. Urgent coronagraphy showed a normal angiogram. Cardiac surgery included aortic valve replacement (mechanical aortic valve ATS No.24 ATS Medical, Inc, Minneapolis, MN, USA), mitral valve annuloplasty (SJM™ Rigid Saddle Ring No 28) (St. Jude Medical, Inc., St. Paul, MN, USA), an excision of tricuspid valve vegetation, triangular resection of the septal leaflet, direct reconstruction with polypropylene 5-0 stitch and De Vega annuloplasty tricuspid annulus reconstruction. Additionally, the excision of vegetation positioned within the posteromedial and posterolateral pulmonary valve leaflet with pulmonary valve plastics and stitches on the pulmonary valve commissures were performed. After 43 days the patient was discharged and was doing well at 4-year follow-up.

The patients’ findings compared to other PVIE cases are shown in Table 1.

## 3. Discussion

Our case series demonstrates that PVIE is usually seen in younger patients. Two of the three had a history of congenital heart disease. The third case was without previous medical history but with IE of three valves. These findings are in line with previous reports that PVIE is usually seen in previous CHD and that structurally normal pulmonary valve is hardly affected alone [11]. Our case series demonstrates that often, signs and symptoms of PVIE can be versatile, and the diagnosis is often delayed. Guidelines recommend that IE should be considered in any patients with CHD presenting with ongoing fever and signs of systemic infection [10]. All cases were associated with serious complications, and one had an unfavorable outcome. Despite improvements in diagnosis and treatment, infective endocarditis is associated with high mortality and severe complications, and remains a therapeutic challenge [12].

Echocardiography is the cornerstone of the diagnostic algorithm in IE. Although echocardiography in PVIE could be challenging for TTE and TOE, both provide fundamental and reliable morphological and functional information for diagnosis [6]. In all three PVIE cases, TTE and TOE were key in confirming the diagnosis. Repeated echocardiography in our cases was useful in complications, clinical follow-up and surgery planning timing.

The microbiology of IE from *Streptococci* has been shifted to *Staphylococci*, these being the most commonly encountered microorganism [13]. Two of our patients’ positive blood cultures yielded a *Staphylococci* species. *Corynebacteria* usually have low virulence and cause catheter-related bloodstream infections and native- and prosthetic-valve endocarditis [14]. *Corynebacteria* were found in our Case No 1 despite no catheter-related involvement, complicating *Staphylococcus* infection. *Klebsiella pneumonia* most often causes life-threatening community- or hospital-acquired pneumonia, but is a rare cause of IE [15,16]. In our Case No 3, *Klebsiella pneumoniae* infection was associated with prolonged antibiotic treatment in secondary care hospital. Culture-negative IE has been connected with increased short-term and long-term mortality, particularly in receiving medical treatment only, similar to in our second case [17].

Although RSIE and PVIE are usually treated conservatively and considered to have a benign course with good clinical outcomes, our case series demonstrates that the clinical course might be very complicated, with an uncertain outcome. In hemodynamic deterioration, embolic events or uncontrolled infection treatment courses should be reconsidered and directed toward cardiac surgery. In the case of our two patients with favorable outcomes, the complicated PVIE course was resolved with urgent cardiac surgery. The presence of ventricular septal defects can lead to the multivalvular distribution of endocarditis [18]. In Case No 2, despite no visible involvement of left-sided structures, the patient experienced systemic embolization, probably due to the right shunt through VSD. The use of TOE is essential in the visualization of structures and intracardiac defects when evaluating a patient with suspected or known endocarditis [19].

The current guidelines define the criteria for surgical intervention for RSIE [10], but those recommendations are mainly based on tricuspid valve involvement studies. Decisions on urgent surgery in critically ill patients with MOF may be debatable [20]. PVIE is very rare; Chowdhury et al. [21] anticipated that 70 cases of isolated PVIE were reported between 1979 and 2013, and among these such devastation of the pulmonary valve and neighboring structures like in the cases we reported are rarely seen.

Expert opinion is that the majority of PVIE could be managed conservatively with lower mortality compared to left-sided infective endocarditis [10,22]; however, as this is a rare form of IE, the evidence for these recommendations is limited. Early reports found that 40% of patients with isolated PVIE eventually required surgery, and the overall mortality was 24% [5], with excellent outcomes in those who underwent surgery. Isaza et el. [23] reported 24 PVIE cases over a 16-year period, and found that 54.2% were with prosthetic valves and 75% required surgery; in hospital, mortality was 8.8% with an overall 2.8-year median follow-up mortality of 41.7%. The authors demonstrated that surgery plays an important role in the management of PVIE, particularly for those with prosthetic PVIE. In addition, the authors reported [24] 12 patients with PVIE; 4 underwent surgery, and 7 were treated conservatively, while mortality was 42.8% in the conservative vs. 25% in the surgery treatment group. In a case series of eight patients [25], five of the six treated conservatively died, and two were operated on with successful outcomes. Also, four cases of PVIE without underlying heart diseases who required surgical interventions [26] exhibited good outcomes. Zhang et al. [27] showed an efficaciously operated case of PVIE, and other authors have documented successfully treating PVIE using pulmonary valve replacement [28,29]. Agrawal et al. [30] reported the successful replacement of the pulmonary valve with a bioprosthetic valve and patch closure of the ASD in patient with PVIE. In an opposite case report, autopsy showed a congenital bicuspid pulmonary valve, affected by infective endocarditis [31], and even after this, a case of cardiac surgery-complicated [32] multi-organ failure concluded with mortality. Dhakam et al. [33] reported a case of PVIE treated conservatively with lethal outcome after prolonged hospital stay.

We encountered several problems in patients with PVIE: the vegetations were multiple and widely spread; thromboembolic complications were present, as well as the MOF; the infective agent was uncommon. In these circumstances, medical therapy is insufficient, and the first-line therapy and approach should be shifted to surgical treatment. Surgical correction with homograft and PV correction could offer a good long-term result. Moreover, to successfully manage PVIE, it is necessary to anticipate possibilities for adverse outcomes [34] using all the therapeutic possibilities in nearby regions with the help of a coordinated ET approach.

## 4. Conclusions

In conclusion, our case series demonstrate that often, the signs and symptoms of this rare form of pulmonary valve endocarditis can be unspecific, versatile, and the diagnosis is often delayed. A high level of suspicion and early echocardiography, especially in patients with congenital heart disease, might be crucial in optimal management. Our experience suggests that an individual approach and cardiac surgery could ensure successful long-term outcome.

## Figures and Tables

**Table 1:** Comparative findings among PVIE cases.

	Case 1	Case 2	Case 3
Age	36	38	41
Medical history	Congenital pulmonary stenosis	Tetralogy of Fallot	Native artery
Clinical presentation	Fever, skin hemorrhage, HF, MOF, sepsis, anemia	Fever, HF, MOF, sepsis	HF, MOF, sepsis
Other valves included	NO	NO	YES (aortic, tricuspid)
TTE	YES	YES	YES
TOE	YES	YES	YES
MSCT	YES	NO	YES
Blood Culture	Positive: *Staphylococcus*, *Corynebacterium*	Negative	Positive: *Staphylococcus* *Klebsiella pneumoniae*
Initial antibiotic treatment	vancomycin ciprofloxacin	ceftriaxone linezolid gentamicin metronidazole	vankomycin gentamycin
ET approach:	YES International	YES	YES
Surgery	YES PV homograft	NO	YES Reconstruction of PV
Clinical Outcome	Cured 5 year f-up	Death 12th day after the admission	Cured 4-year f-up

Legend: ET—endocarditis team, HF—heart failure, MOF—multiorgan failure, MSCT—multi-slice computed tomography; PV—pulmonary valve, TTE—transthoracic echocardiography; TOE—transesophageal echocardiography.

## Supplementary Materials

The following supporting information can be downloaded at: https://www.mdpi.com/article/10.3390/medicina59071213/s1, Video S1: Vegetations on PV and RVOT; Video S2: Pulmonary valve vegetation.
